# Caveolin-1 mediates the utilization of extracellular proteins for survival in refractory gastric cancer

**DOI:** 10.1038/s12276-023-01109-7

**Published:** 2023-11-02

**Authors:** Nahee Hwang, Bo Kyung Yoon, Kyu-Hye Chun, Hyeonhui Kim, Yoseob Lee, Jae-Won Kim, Hyeonuk Jeon, Tae-Hyun Kim, Mi-Young Kim, Sungsoon Fang, Jae-Ho Cheong, Jae-woo Kim

**Affiliations:** 1 Department of Biochemistry and Molecular Biology, Yonsei University College of Medicine, Seoul, Republic of Korea; 2 Graduate School of Medical Science, Brain Korea 21 Project, Yonsei University College of Medicine, Seoul, Republic of Korea; 3 Chronic Intractable Disease for Systems Medicine Research Center, Yonsei University College of Medicine, Seoul, Republic of Korea; 4 Severance Biomedical Science Institute, Gangnam Severance Hospital, Yonsei University College of Medicine, Seoul, Republic of Korea; 5 Department of Surgery, Yonsei University College of Medicine, Seoul, Republic of Korea; 6 Department of Biomedical Systems Informatics, Yonsei University College of Medicine, Seoul, Republic of Korea; 7 Department of R&D, Veraverse Inc., Seoul, Republic of Korea

**Keywords:** Gastric cancer, Cancer metabolism, Cancer therapeutic resistance

## Abstract

Despite advances in cancer therapy, the clinical outcome of patients with gastric cancer remains poor, largely due to tumor heterogeneity. Thus, finding a hidden vulnerability of clinically refractory subtypes of gastric cancer is crucial. Here, we report that chemoresistant gastric cancer cells rely heavily on endocytosis, facilitated by caveolin-1, for survival. caveolin-1 was highly upregulated in the most malignant stem-like/EMT/mesenchymal (SEM)-type gastric cancer cells, allowing caveolin-1-mediated endocytosis and utilization of extracellular proteins via lysosomal degradation. Downregulation of caveolin-1 alone was sufficient to induce cell death in SEM-type gastric cancer cells, emphasizing its importance as a survival mechanism. Consistently, chloroquine, a lysosomal inhibitor, successfully blocked caveolin-1-mediated endocytosis, leading to the marked suppression of tumor growth in chemorefractory gastric cancer cells in vitro, including patient-derived organoids, and in vivo. Together, our findings suggest that caveolin-1-mediated endocytosis is a key metabolic pathway for gastric cancer survival and a potential therapeutic target.

## Caveolin-1: Survival Mechanism in Gastric Cancer Cells

A new study reveals that Caveolin-1 (CAV1) is specifically expressed in the most malignant SEM-type gastric cancer (GC), and CAV1-driven endocytosis is a critical survival mechanism of malignant GC. Researchers found that chloroquine (CQ), an anti-malarial drug known to block endocytosis, effectively induced cell death in CAV1-positive SEM-type GC cells by impairing lysosomal activity and extracellular nutrient uptake. In patient-derived organoids and mouse models, CQ treatment significantly reduced tumor growth and improved survival rates, suggesting its potential as a therapeutic alternative for chemo-resistant SEM-type GC patients. The study highlights the importance of targeting endocytosis in cancer treatment and provides new insights into the development of personalized therapies for GC patients.

## Introduction

Gastric cancer (GC) exhibits high degrees of intertumoral heterogeneity and lineage diversity, resulting in inconsistent responses to anticancer treatments^1^. Therefore, classifying GC subtypes based on their molecular characteristics is necessary to enable individualized treatments. In a previous study, we classified GC into five subtypes (gastric, mixed, inflammatory, intestinal, and stem-like) based on transcriptomic profiles from a large pool of patients. Among these subtypes, the stem-like subtype exhibits the highest chemoresistance, leading to the worst prognosis^1^. Although chemoresistance is well known, there are no alternative treatment options available for stem-like subtype GC patients. Therefore, we focused on providing therapeutic alternatives for these patients. Additionally, we found that the stem-like subtype has genetic similarities to the most malignant types identified in other GC cohorts^1^: genome stable (GS) type of The Cancer Genome Atlas Stomach Adenocarcinoma (TCGA-STAD), epithelial–mesenchymal transition (EMT) type of Asian Cancer Research Group (ACRG), and mesenchymal type classified by Lei et al. ^2^. In this study, we integrated these malignant GC types with similar characteristics by designating them as the SEM (stem-like, GS, EMT, and mesenchymal) type.

Endocytosis is the process of internalization of the plasma membrane and its components into cells^3^. Once inside the cell, these components undergo degradation via the lysosome-dependent pathway. Lysosomes contain various hydrolytic enzymes that break down biological compounds, such as proteins, lipids, and nucleic acids, to produce energy and support anabolic processes^3–5^. Because tumor cells are exposed to a metabolically stressful environment in vivo, targeting the endocytosis-lysosome-mediated pathway is considered a new therapeutic approach^6–10^. Additionally, endocytosis can be broadly classified into four categories: phagocytosis, macropinocytosis, clathrin-mediated endocytosis, and caveolae-mediated endocytosis, based on their distinct shape, size, and constituent proteins. Among these categories, caveolae-mediated endocytosis is associated with the internalization of albumin and is mainly regulated by the protein caveolin-1 (CAV1)^3,11,12^.

The role of CAV1 in cancer cells is still controversial^13–19^. Although CAV1 has been known to be upregulated in cancer cells^20,21^, there are reports that CAV1 is downregulated in lung, breast, colon, and ovarian cancer^22,23^. In breast cancer, CAV1 has been shown to inhibit the growth and metastasis of cancer cells and suppress the self-renewal capacity of cancer stem cells^19,24^. However, in colon cancer, the re-expression of CAV1 blocked tumor formation. This highlights the need to clarify the function of CAV1 in chemorefractory GC. In this study, we revealed that CAV1 is specifically expressed in the most malignant SEM-type GC, and CAV1-driven endocytosis is a critical survival mechanism of malignant GC.

Considering endocytosis as a potential “Achilles’ heel” of GC cells, we applied chloroquine (CQ), an antimalarial drug with the ability to block endocytosis^25,26^, as a treatment. CQ is known as an autolysosomal inhibitor, a p53-pathway activator, and an apoptosis inducer in pancreatic cancer, lymphoma, and glioma, respectively^27–29^. Although there have been several clinical trials that attempted to repurpose CQ as an anticancer drug, none have been successful^30–32^. We speculate that the causes of clinical failure may arise from intertumoral heterogeneity. Indeed, the selection of a patient subgroup with stricter standards, predicted to be responsive to the intended treatment, is essential for the successful clinical evaluation of an investigational drug. Accordingly, we also focused on providing biomarkers for predicting the drug response to CQ based on the molecular mechanisms of SEM-type GC-specific efficacy.

Here, we report that CAV1 is highly expressed only in the SEM type among GC subtypes. Confocal imaging of CAV1 shows its abundant presence in lysosomes, providing a new perspective on CAV1-mediated endocytosis as a critical survival mechanism for chemorefractory GC, which is prone to metastasis. Finally, our results demonstrate the effectiveness of CQ treatment in SEM-type GC in patient-derived organoids and mouse models, shedding light on therapeutic alternatives for clinically intractable cancers.

## Materials and methods

### Clinical cohort

We received frozen tissue and clinical data of patients who received curative intent gastrectomy at Yonsei Cancer Center (Seoul, Republic of Korea). The present study was approved by the institutional review board of Severance. All samples were collected after obtaining written informed consent from the patients. Using samples, we generated two batches of cohort datasets (*n* = 497; GSE13861 and GSE84437; Illumina HUmanHT-12 v3.0 Expression BeacChip array).

### Cell culture

HS746T cells were cultured in DMEM containing 10% FBS, 2 mM L-glutamine, and 100 U/ml penicillin/streptomycin. SNU668, MKN1, SNU601, KATOIII, and NCIN87 cells were cultured in RPMI 1640 containing 10% fetal bovine serum (FBS), 2 mM L-glutamine, and 100 U/ml penicillin/streptomycin. All cells were cultured at 37 °C in a water-saturated incubator with 5% CO2 and were tested for mycoplasma contamination.

### Organoid culture

Organoids were generated based on a previous study with a slight alteration^33^. The gastric fundus organoids were taken from surgical samples of GC patients at Yonsei University Severance Hospital who had given prior consent (IRB No. 4-2017-0106). The organoids were cultured in Matrigel (BD Bioscience, USA) and medium in a 48-well plate. The medium composition was as follows: advanced Dulbecco’s modified Eagle’s medium/F12 medium (Invitrogen, USA), Wnt-conditioned medium, and R-spondin-conditioned medium supplemented with gastric growth factors, including BMP inhibitor, noggin (PeproTech, USA), GlutaMAX-I (Invitrogen, USA), B27 (Invitrogen, USA), TGF beta I A83-01 (TOCRIS, UK), nicotinamide (Sigma, USA), *N*-acetylcysteine (Sigma, USA), gastrin (Sigma, USA), EGF (PeproTech, USA), and FGF10 (R&D Systems, USA). Organoids were formed within 1–2 days and passaged every 7 days. To evaluate the effect of CQ, each group of organoids (*n* = 3/group) was cultured with/without 50 µg/ml CQ, and the diameter was measured on the first and fifth days of treatment.

### Tumor xenograft mouse models

Mice were acclimated for 7 days under a 12 h light/12 h dark cycle. Xenograft models were generated by injecting 5 × 10^5^ HS746T cells with 50% Matrigel (Corning, USA) into the flank of BALB/c-nude mice. After 3 days, mice were randomly divided into 3 groups (*n* = 10/group) for saline or CQ treatment. Tumor volume was measured when it reached ~100 mm^3^, and mice were randomly divided into 2 groups (*n* = 9/group) for daily CQ or saline injections for 3 weeks. Tumor volume was calculated using (long diameter × short diameter^2^)/2. After the injection period, the mice were sacrificed, and the tumors were harvested. For iPM mouse models, mice were injected with HS746T-GFP cell lines diluted in DMEM in a total volume of 100 ml. After 7 days, mice were randomly divided into 2 groups (*n* = 10/group) for daily saline or 60 mg/kg CQ injections for 3 weeks.

### Statistical analyses

One-way ANOVA was used to test for differences among three or more groups, while Student’s *t* test was used to compare two groups. Statistical significance was defined as *p* < 0.05 (*p < 0.05, **p < 0.01, ***p < 0.001), with means and standard errors of the mean presented for all values.

Please see the Supplementary information for detailed information on the materials and processes used in this study.

## Results

### CAV1 is highly enriched in chemorefractory SEM-type GC

**Fig. 1:**
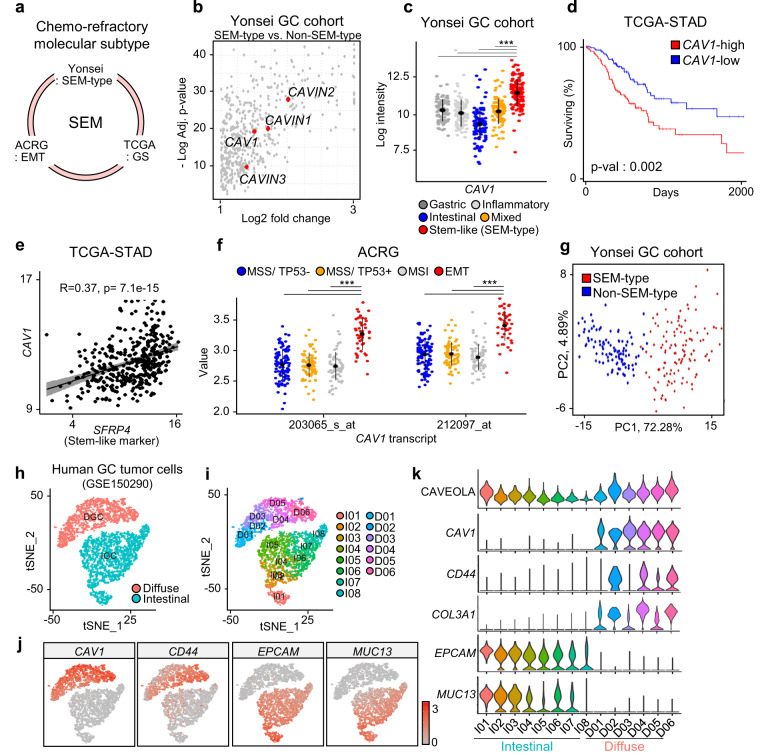
CAV1 is highly enriched in patients with chemorefractory SEM-type GC. **a** A diagram of the most malignant subtype of GC patients in multiple cohorts (Yonsei GC cohort, TCGA-STAD, and ACRG cohorts). **b** Transcriptome data on tumors of GC patients (*n* = 497) in the Yonsei cohort were analyzed. Volcano plot based on upregulated differentially expressed genes (log2-fold change >1, adjusted *p* value < 0.05) of the stem-like subtype compared to the intestinal subtype. **c** The expression level of *CAV1* was compared according to the subtype: mixed (*n* = 99), gastric (*n* = 89), mesenchymal (*n* = 117), intestinal (*n* = 102), and inflammatory (*n* = 90). **d** Kaplan–Meier plot for the CAV1-high group (*n* = 113) and CAV1-low group (*n* = 133) in TCGA-STAD patients Logrank = 0.002. **e** Pearson correlation between *SFRP4* and *CAV1* in TCGA STAD (*n* = 375) *R* = 0.37, *p* = 7.1 × 10-15. **f** Transcriptome data on tumors of GC patients (*n* = 300) in the ACRG cohort (GSE66229) were analyzed. The expression levels of *CAV1* transcripts were compared according to the subtype: EMT (*n* = 46), MSI (*n* = 68), MSS/TP53+ (*n* = 107), and MSS/TP53- (*n* = 79). **g** Principal component analysis (PCA) based on coexpression genes of *CAV1* from stem-like and intestinal subtype GC patients. Genes coexpressed with *CAV1* were analyzed from the SEEK gastric cancer dataset. **h**, **i** Uniform manifold approximation projection (UMAP) plot of 7,550 tumor cells in GC tumors. Colors represent cell types based on the expression of marker genes. **j** UMAP plot showing normalized expression of *CAV1, CD44, EPCAM and MUC13*. **k** Violin plots showing normalized expression of genes and enrichment score of the CAVEOLA pathway in intestinal and diffuse types of GC clusters. Data represent the mean ± SD. ***p < 0.001; two-tailed *t* test.

To determine potential therapeutic targets for the most malignant type of GC, we first conducted a transcriptomic analysis on GC patients across multiple cohorts (Yonsei GC cohort, TCGA-STAD, and ACRG cohorts). Previously, we reported that the SEM type (*n* = 117) of the Yonsei GC cohort data (*n* = 497), the genome-stable (GS) type of TCGA-STAD data, the epithelial–mesenchymal transition (EMT) type of the ACRG cohort data, and the mesenchymal type classified by Lei et al. ^2^ share similar molecular and genetic characteristics, as well as clinical prognoses^1^. In this study, we comprehensively named the most malignant types of GC the SEM type (Fig. 1a). Based on the Yonsei GC cohort, we found that *CAV1* was one of the top genes highly enriched in the most malignant SEM type of GC compared to the intestinal epithelial GC type (non-SEM), whose genetic and molecular characteristics are the most distinct from SEM (Fig. 1b, c). Moreover, the expression of *CAV1* was negatively correlated with the overall survival of GC patients in the TCGA gastric cancer database (Fig. 1d). *CAV1* was also positively correlated with secreted frizzled-related protein 4 (*SFRP4*), a clinical marker of SEM-type GC^1^. (Fig. 1e). Similar to the findings from the Yonsei GC cohort, *CAV1* was markedly increased in EMT-type GC compared with non-EMT-type GC in the ACRG cohort (Fig. 1f). Next, we performed a principal component analysis on patients with SEM and intestinal-type GC based on 100 genes that had the highest coexpression score with *CAV1* according to the SEEK database (37 human GC public datasets). Interestingly, the SEM and non-SEM types of GC patients were distinctly located in different clusters, indicating the possibility of *CAV1* as a major biomarker of SEM-type GC (Fig. 1g). As SEM-type GC is known to be highly related to Lauren’s diffuse subtype^1^, we compared the expression of *CAV1* between diffuse- and intestinal-type GC tumors. We analyzed single-cell RNA sequencing data of tumor samples from 24 GC patients (GSE150290)^34^. After quality control, a total of 7550 tumor cells were classified into diffuse and intestinal types according to the expression of previously provided marker genes for the intestinal type (*EPCAM, MUC13*, and *COL3A1*)^34^ and diffuse type (cancer stem cell marker *CD44*)^35^ (Fig. 1h-k) and 11 clusters according to their transcription characteristics (Fig. 1i). Interestingly, the expression of CAV1 was significantly enriched in the diffuse type of GC clusters (Fig. 1j, k). In addition, the enrichment score of the CAVEOLA pathway was significantly high in diffuse-type GC clusters (Fig. 1k). These results highlight CAV1 as a biomarker for SEM-type GC patients.

### CAV1 plays a critical role in the survival of SEM-type GC cells

**Fig. 2:**
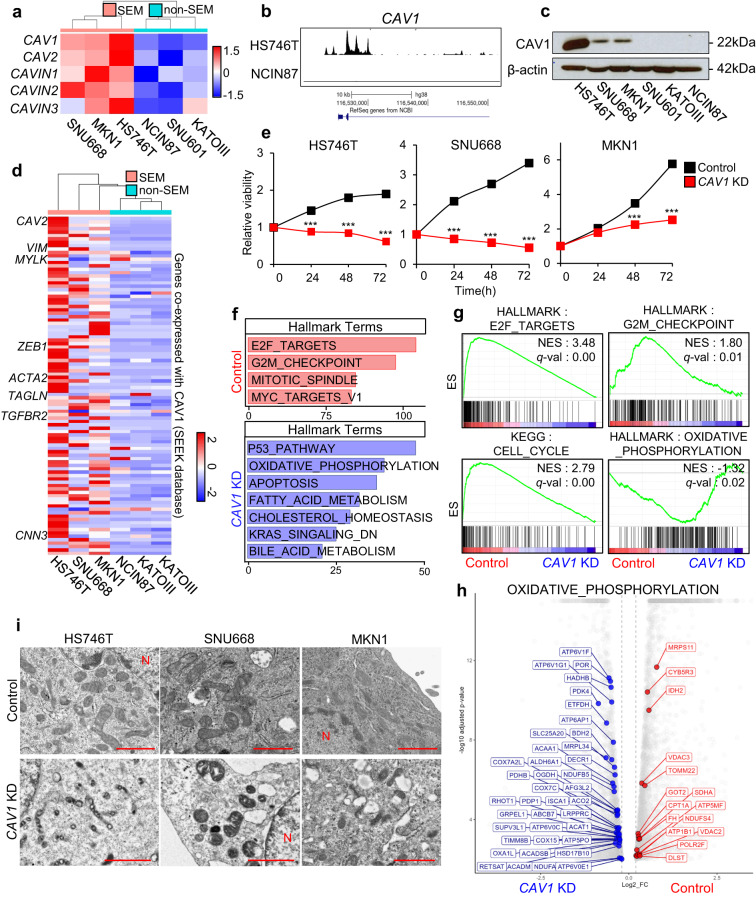
CAV1 plays a critical role in the survival of SEM-type GC cells. **a** A heatmap of caveolae component genes in GC cell lines. SEM-type GC cells HS746T, MKN1, SNU668 and non-SEM-type GC cells SNU601, KATOIII, NCIN87. **b** Representative sequencing tracks for the *CAV1* locus show distinct ATAC-seq peaks at the promoter in GC cells. The ATAC-seq data were normalized to take sequencing depth into account, and the scale on the y-axis was chosen for optimal visualization of peaks for each sample. **c** Protein levels of CAV1 in GC cells. **d** A heatmap of the expression levels of *CAV1* coexpressed genes in GC cell lines. **e** Relative viability of SEM-type GC cells after *CAV1* knockdown (*CAV1* KD, *n* = 3/group). Cell proliferation was measured by Adam at various times. Cell numbers are presented as the percentage of cells at 0 h. **f** The top enriched pathways of differentially expressed genes (DEGs) in *CAV1*-knockdown HS746T cells were analyzed by enrichR with the MSigDB_Hallmark_2020 library (red; upregulated in control cells, blue; upregulated in *CAV1*-KD cells). **g** Gene set enrichment analysis (GSEA) for HALLMARK_E2F_TARGETS, HALLMARK_G2M_CHECKPOINT, KEGG_CELL_CYCLE and HALLMARK_OXIDATIVE_PHOSPHORYLATION from MSigDB v7.0 in control and *CAV1*-knockdown HS746T cells. **h** Volcano plot representing gene sets of HALLMARK_OXIDATIVE_PHOSPHORYLATION in the DEGs of HS746T cells after *CAV1* knockdown (blue; downregulated, red; upregulated, log2-fold change >0.2, adjusted *p* value < 0.01). **i** TEM images showing damaged mitochondria in *CAV1*-knockdown HS746T cells. N; nucleus, arrow; mitochondria. Scale bar, 1 µm. Data represent the mean ± SD. *** adjusted p-value < 0.001; Benjamini‒Hochberg procedure.

To verify our findings in vitro, we evaluated whether CAV1 was enriched in SEM-type GC cell lines grouped by transcriptomic characteristics^36^. The expression of CAV1 and other genes that constitute caveolae was markedly elevated in SEM-type GC cell lines compared to non-SEM-type GC cell lines (Fig. 2a). We compared chromatin accessibility at the *CAV1* promoter in HS746T and NCIN87 cells (representing SEM-type and non-SEM-type GC cell lines, respectively) by analyzing transposase-accessible chromatin using sequencing data. As expected, chromatin accessibility increased significantly only in HS746T (Fig. 2b). SEM-type GC cell lines showed much higher protein levels of CAV1 than non-SEM-type GC cell lines (Fig. 2c). Genes coexpressed with CAV1, which are related to a mesenchymal-like feature, were upregulated in SEM-type GC cell lines (Fig. 2d). Since SEM-type GC cells featured chemoresistance (Supplementary Fig. 1a), we explored the relationship between CAV1 expression and cisplatin responsiveness. The expression of CAV1 was increased by cisplatin treatment in the SEM-type GC cell lines MKN1 and SNU668 (Supplementary Fig. 1b). These findings suggest that CAV1 has functional implications in chemorefractory SEM-type GC cells.

To verify the role of CAV1 in SEM-type GC cells, we knocked down CAV1 in SEM-type GC cells (Supplementary Fig. 1c, d). Interestingly, knockdown of *CAV1* effectively inhibited the growth of SEM-type GC cells and eventually led to cell death, indicating the critical roles of CAV1 in their survival (Fig. 2e, Supplementary Fig. 1e). Next, we performed bulk RNA sequencing with *CAV1*-knockdown SEM-type GC cells, which showed severe downregulation of genes involved in the cell cycle and upregulation of genes involved in apoptosis (Fig. 2f, g and Supplementary Fig. 2a). Additionally, there were significant changes in metabolic pathways in SEM-type GC cells, especially oxidative phosphorylation (Fig. 2f-h). Transmission electron microscopy (TEM) images showed that *CAV1* knockdown triggered severe damage to mitochondria in SEM-type GC cells (Fig. 2i and Supplementary Fig. 2b).

### CAV1 mediates endocytosis to utilize extracellular proteins in SEM-type GC cell lines

**Fig. 3:**
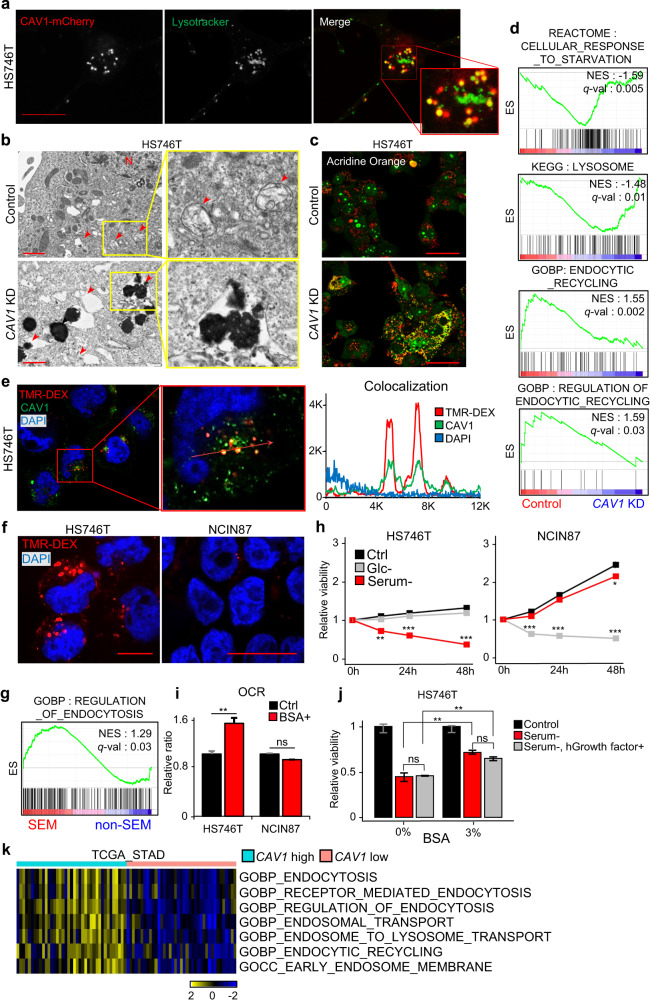
CAV1 mediates endocytosis to utilize extracellular proteins in SEM-type GC cells. **a** Live cell images of HS746T cells expressing mCherry-CAV1 (*left*) and LysoTracker (*middle*). Merged images (mCherry-CAV1; green, LysoTracker; red, *right*). Scale bar 10 µm. **b** TEM images showing damaged lysosomes in SEM-type GC cells with CAV1 knockdown. N; nucleus, arrow; mitochondria. Scale bar, 1 µm. **c** Confocal images of *CAV1*-knockdown HS746T cells stained with acridine orange. Scale bar, 50 µm. **d** RNA-seq data of *CAV1*-knockdown HS746T cells were analyzed. GSEA for KEGG_LYSOSOME, REACTOME_CELLULAR_RESPONSE_TO_STARVATION, GOBP_ENDOCYTIC_RECYCLING, and GOBP_REGULATION_OF_ENDOCYTIC_RECYCLING from MSigDB v7.0. **e** Colocalization of CAV1 (green) and TMR-DEX (red) in HS746T cells. Scale bars, 20 µm. **f** Confocal images of HS746T and NCIN87 cells after the TMR-DEX uptake assay. Scale bars, 10 µm. **g** GSEA for GOBP _REGULATION_OF_ENDOCYTOSIS for SEM-type GC cell lines compared to non-SEM-type GC cell lines. **h** Relative survival ratio of HS746T and NCIN87 cells cultured in nutrient-deprived conditions (glucose and serum, respectively). **i** OCR ratio of HS746T and NCIN87 cells cultured in BSA with/without treatment (*n* = 3/group). The result is presented as the percentage of OCR (pmol/min) in the control group. **j** HS746T cells cultured in serum-free media +/- human recombinant growth factors (Rocket fuel) with/without 3% BSA (*n* = 3/group). Total cell numbers were measured by CCK-8 assay and presented as the percentage of cells in the control group. **k** A heatmap of GSVA of endocytosis-related pathways from MSigDB v7.0 between the CAV1-high and -low groups in TCGA-STAD data. Patients were classified according to the RNA expression levels of *CAV1* (*n* = 40/group). Data represent the mean ± SD. *p < 0.05; **p < 0.01; ***p < 0.001; two-tailed *t* test.

While CAV1 is a well-known membranous protein, immunofluorescence images revealed that it was abundantly present in the cytoplasm as well as the cellular membrane of SEM-type GC cells (Supplementary Fig. 3a). Cytoplasmic Caveolin-1 is known to be in the membranes of diverse organelles, such as mitochondria, endoplasmic reticulum (ER), Golgi apparatus, and lysosomes^37,38^. For further analysis, live cell imaging was performed after staining the mitochondria, ER, and lysosomes. Interestingly, while CAV1 was not present in either mitochondria or the ER (Supplementary Fig. 3b, c), it was found in lysosomes of SEM-type GC cells (Fig. 3a and Supplementary Fig. 3d). We tested whether inhibition of CAV1 changed lysosomal activity. To visualize lysosomes after *CAV1* knockdown, TEM imaging and acridine orange staining were conducted. Inhibition of *CAV1* remarkably induced lysosomal defects with an increase in the pH value (Fig. 3b, c and Supplementary Fig. 4a). Moreover, *CAV1* knockdown significantly changed the expression of genes involved in lysosomes in SEM-type GC cells (Fig. 3d). Additionally, *CAV1* knockdown upregulated the cell starvation pathway, while the endocytic recycling pathway was markedly downregulated in SEM-type GC cell lines (Fig. 3d). In addition, live imaging of CAV1-overexpressing SEM-type GC cells revealed the dynamic internalization of membranous CAV1 into the cytoplasm, specifically at the site of cellular pods during active movement. This observation suggests an association between cell migration and endocytosis via CAV1 (Supplementary Video 1). These results suggest that CAV1 may be associated with endocytosis, which is mediated by lysosomes^39,40^ in SEM-type GC cells. Endocytosis is one of the important strategies used by cancer cells to take up extracellular macromolecules as nutrients for their survival in a metabolically stressful microenvironment^8–10^. For example, pancreatic ductal adenocarcinoma activates endocytosis to take up external nutrients and maintain the cellular amino acid level required for survival^41^. To determine whether CAV1 modulates endocytosis in SEM-type GC cells, we conducted a tetramethyl rhodamine-labeled high-molecular mass dextran (TMR-DEX) uptake assay^42,43^. We observed that CAV1 colocalized with endocytic dextran in SEM-type GC cells (Fig. 3e). Furthermore, transient knockdown of *CAV1* significantly reduced endocytosis-mediated dextran uptake (Supplementary Fig. 4b), whereas *CAV1* overexpression markedly increased dextran uptake in SEM-type GC cells (Supplementary Fig. 4c), suggesting that CAV1 plays a crucial role in the uptake of extracellular macromolecules. Moreover, SEM-type GC cells exhibited a higher endocytosis rate than CAV1-negative non-SEM-type GC cells (Fig. 3f and Supplementary Fig. 4d, e), indicating that CAV1 may modulate endocytosis in SEM-type GC cells. Additionally, the regulation of endocytosis was enriched in SEM-type GC cells (Fig. 3g). To examine the dependence of extracellular macromolecules as energy sources, we performed a cell viability assay in the absence of glucose and serum. Interestingly, serum starvation markedly decreased cell viability in SEM-type GC cells; however, glucose starvation affected cell viability to a greater degree in non-SEM-type GC cells than in SEM-type GC cells (Fig. 3h). Treatment with bovine serum albumin (BSA) also significantly increased the oxygen consumption rate in SEM-type GC cells (Fig. 3i). Moreover, the addition of BSA effectively improved the viability of SEM-type GC cells in the presence of serum deprivation. On the one hand, the addition of Rocket Fuel-R™, which contains recombinant human growth factors, only minimally improved the serum deprivation-induced cell death of SEM-type GC cells (Fig. 3j). Furthermore, the addition of lipid-free BSA also resulted in the recovery of cell viability, thus excluding the influence of lipids (Supplementary Fig. 4f). To investigate the relationship between CAV1 expression and the survival of albumin-dependent SEM-type GC cells, we cultured CAV1 knockdown cells under various conditions, including control, 1% FBS media with low glucose (1.0 g/L), high glucose (4.5 g/L), low glucose + 3% BSA, and low glucose + 300 µM oleic acid (Supplementary Fig. 4g). The decreased cell viability of si-control cells in 1% FBS was significantly rescued only in the low glucose + 3% BSA condition. On the other hand, in CAV1 knockdown cells, it was evident that cell survival decreased regardless of the additional nutrient supply. These results demonstrate that the death of SEM-type GC cells in the absence of serum is not caused by growth factors and lipids but by albumin. CAV1 is crucial for the acquisition of extracellular proteins, which serve as the primary energy source. These results demonstrate that the death of SEM-type GC cells in the absence of serum is not caused by growth factors but by albumin. They also show that extracellular proteins are the main energy source required for the survival of SEM-type GC cells. Finally, the Gene set variation analysis enrichment score for endocytosis-related gene signatures was markedly enriched in the *CAV1*-high patient group in the TCGA-STAD data (Fig. 3k).

### CQ, an endocytosis-blocking agent, has an anticancer effect in SEM-type GC cells and mouse xenograft models

**Fig. 4:**
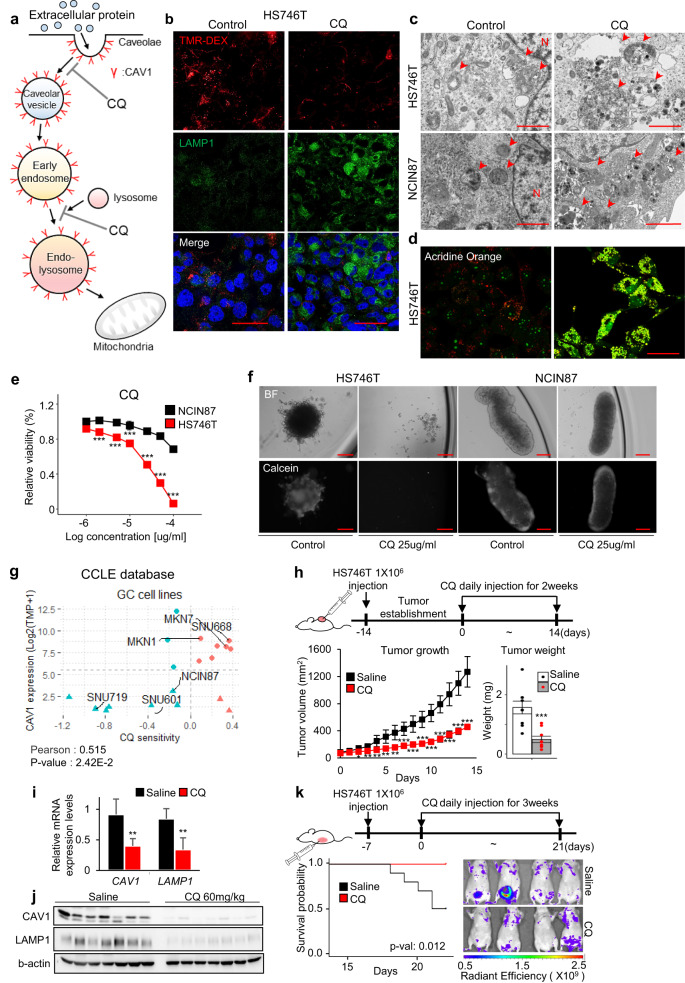
CQ, an endocytosis-blocking agent, has an anticancer effect in SEM-type GC cells and mouse xenograft models. **a** A model showing the mechanism of CQ to suppress CAV1-mediated (**b**) TMR-DEX uptake assay for HS746T after CQ treatment. Absorbed cellular TMR-DEX (red) and LAMP1 (green) are shown. Scale bars, 40 µm. **c** TEM images showing damaged lysosomes in CQ-treated HS746T and NCIN87 cells. N; nucleus, arrow; lysosome. Scale bar, 1 µm. **d** Confocal images of CQ-treated HS746T cells stained with acridine orange. Scale bar, 50 µm. **e** The IC50 values of CQ treatment for HS746T (red) and NCIN87 (black) cells. **f** Images of HS746T and NCIN87 cells cultured in sphere-forming assay plates and treated with CQ. **g** A scatterplot showing the relationship between the expression level of CAV1 and sensitivity to CQ in GC cell lines. The dataset was from CCLE. **h** HS746T-bearing mice (*n* = 9/group) were injected intraperitoneally with saline or CQ. Tumor volume was measured daily (left), and the final tumor weight was measured at the end of injection (right). **i**, **j** Expression of CAV1 at the RNA (**i**) and protein (**j**) levels in tumors in GC xenograft mouse models injected with CQ. **k** The survival curve of iPM model mice injected with saline or CQ (*n* = 10/group) (left) and IVIS images of tumor burden in surviving mice on the last day of injection (right). Scale bar, 200 µm. Data represent the mean ± SD. **p < 0.01; ***p < 0.001; two-tailed *t* test.

Chloroquine (CQ) is a well-known lysosomal inhibitor and has been shown to impair endocytosis^25,26^. Thus, we next examined the effect of CQ as an anticancer drug against SEM-type GC, which relies on the endocytosis pathway for extracellular nutrients (Fig. 4a). As expected, CQ treatment markedly reduced the uptake of TMR-DEX in SEM-type GC cells (Fig. 4b). Additionally, the effect of CQ, a lysosomotropic agent that induces lysosomal swelling, was confirmed at the protein level of lysosomal-associated membrane protein 1 (LAMP1) (Fig. 4b). TEM and AO staining revealed that lysosomal defects were significantly increased after CQ treatment in both SEM-type and non-SEM-type GC cells (Fig. 4c, d). Although both groups showed severe lysosomal damage, SEM-type GC cells were more susceptible to CQ, hydroxy chloroquine, and mefloquine than non-SEM-type GC cells (Fig. 4e, f and Supplementary Fig. 5a). Moreover, CQ treatment caused a notable shift in the metabolic phenotype of SEM-type GC cells, inducing a transition of the metabolic state from an aerobic to a glycolytic state (Supplementary Fig. 5b). To investigate the association between CAV1 expression and CQ sensitivity, we treated GC cells with CQ after CAV1 knockdown. The significant regression in cell survival in SEM-type GC cells through either CQ treatment or serum deprivation was not observed after CAV1 knockdown (Supplementary Fig. 5c). By analyzing the data of GC cell lines from the CCLE database, we confirmed that there is a positive correlation between *CAV1* expression and CQ sensitivity. Most SEM-type GC cells belonged to a group with high CQ sensitivity (Fig. 4g). These results imply that CQ effectively induces cell death by impairing lysosomal activity and extracellular nutrient uptake by CAV1-positive SEM-type GC cells. Finally, we tested the therapeutic impacts of CQ on tumor growth using a xenograft mouse model. In accordance with the anticancer effect of CQ in SEM-type GC cell lines, tumor growth was markedly reduced by CQ treatment (Fig. 4h). The RNA and protein levels of CAV1 were both significantly decreased in the tumors of the CQ-treated group (Fig. 4i, j). Because peritoneal metastasis is a well-known common characteristic of metastatic GC with poor prognosis^44,45^, we established an intraperitoneal metastasis (iPM) mouse model for survival testing. As expected, CQ injection greatly reduced the tumor burden and increased the survival rate (Fig. 4k). Overall, we demonstrated that CQ treatment was crucial to reduce the tumor burden and improve the survival rate in a xenograft mouse model with CAV1-positive SEM-type GC.

### CQ treatment triggers SEM-type GC organoid regression

**Fig. 5:**
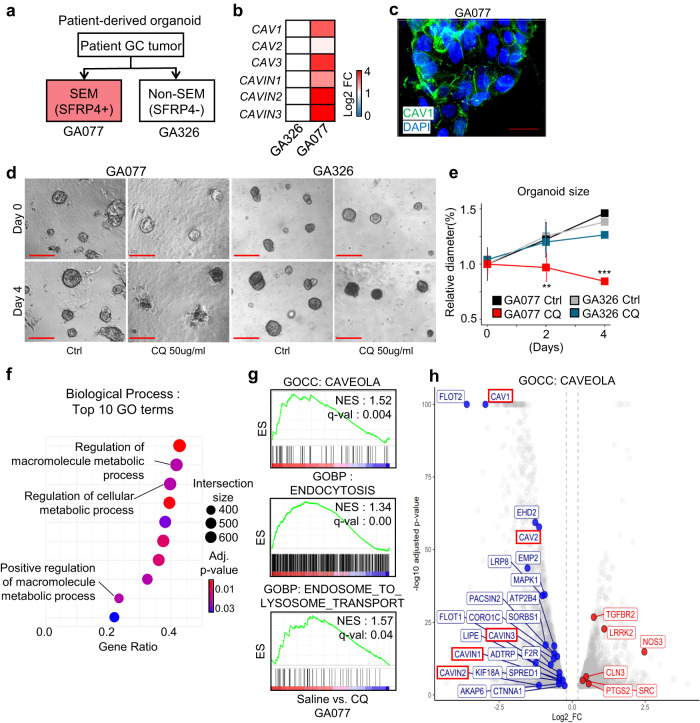
CQ treatment triggers SEM-type GC organoid regression. **a** Diagram explaining the rationale for selecting tumors for the generation of patient-derived organoids. **b** A heatmap of the log two-fold change in *CAV1* and caveolae component genes in SEM-type (GA077) vs. non-SEM-type (GA326) organoids. **c** A 3-dimensional confocal image of CAV1 in GA077 organoids. Scale bars, 40 µm. Organoids with/without CQ (50 µg/ml) treatment (*n* = 6/group). **d**, **e** Microscopy images (**d**) and relative diameter of organoids (**e**). Diameters of each group are presented as the percentage of the diameter on Day 0. Scale bar, 200 µm. **f** Analysis of RNA-seq data of GA077 treated with CQ in terms of GO biological processes. The data show the top 10 GO terms, and dot size represents intersection size. **g** GSEA data of GECC_CAVEOLA (top), GOPB_ENDOCYTOSIS (middle), and GOBP_ENDOSOME_TO_LYSOSOME_TRANSPORT (bottom) for CQ-treated GA077 cells. **h** A Volcano plot representing the gene set of GOCC_CAVEOLA terms in DEGs of CQ-treated GA077 (blue; downregulated, red; upregulated, log two-fold change >0.2, adjusted *p* value < 0.0). Data represent the mean ± SD. **p < 0.01; ***p < 0. 001; two-tailed *t* test.

To verify the anticancer effect of CQ in CAV1-positive SEM-type GC under more physiological conditions, we first generated patient-derived organoids from SFRP4-high (SEM-type) and SFRP4-low (non-SEM-type) tumors from GC patients^1^ (Fig. 5a). Consistent with the in vitro data, CAV1 and caveolae-related genes were upregulated in SEM-type GC organoids (Fig. 5b). Additionally, CAV1 was present in both the membrane and cytoplasm of SEM-type GC organoids (Fig. 5c). The growth of CAV1-positive SEM-type GC organoids was remarkably inhibited by CQ treatment compared to that of the CAV1-negative non-SEM-type group (Fig. 5d, e). Gene ontology analysis indicated that CQ effectively inhibited macromolecule metabolic processes and the endomembrane system of CAV1-positive GC organoids (Fig. 5f). The enrichment scores of GOCC_CAVEOLA, GOBP_ENDOCYTOSIS, and GOCC_EARLY_ENDOSOME were markedly downregulated in CQ-treated SEM-type GC organoids (Fig. 5g). Interestingly, CAV1 was one of the most downregulated genes after CQ treatment (Fig. 5h). In conclusion, we demonstrated that CQ treatment successfully inhibited the endocytosis and growth of CAV1-positive GC organoids.

### Chloroquine effectively targets stemness-high clusters in SEM-type GC organoids

**Fig. 6:**
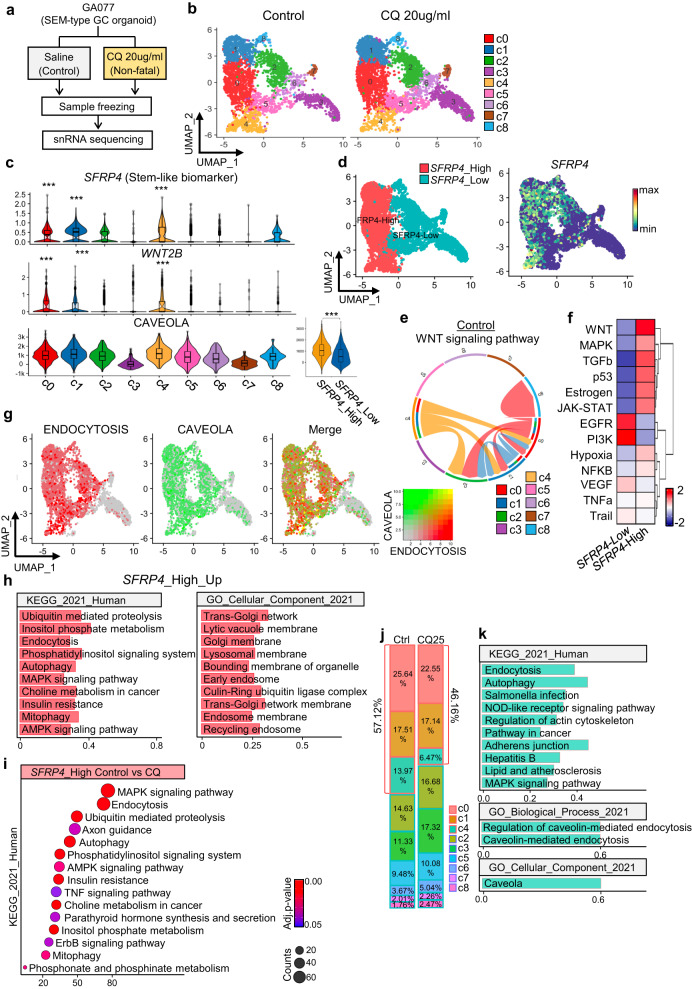
Chloroquine effectively targets stemness-high clusters within SEM-type GC organoids. Single-cell nucleus RNA sequencing was performed on GA077 cells treated with saline or 20 µg/ml CQ for 4 days. **a** Schematic diagram of the sampling process for single-nucleus RNA sequencing. **b** UMAP visualization of cells after integration. **c** The expression levels of *SFRP4* and *WNT2B* and the enrichment score of the CAVEOLA pathway in each cluster are shown as violin plots. **d** UMAP visualization of GC organoids classified by the expression levels of *SFRP4*. **e** Circular plot showing the cell-to-cell network inferred for the WNT signaling pathway of the control sample. The color of the circle varies from cluster to cluster. **f** Heatmap inferring signaling activity for *SFRP4*-high and -low clusters in the control sample. **g** UMAP for each enrichment score and coenrichment score of the endocytosis and caveola pathways in GA077. **h** KEGG and GOCC analysis of DEGs in the *SFRP4*-high cluster compared to the SFRP4-low cluster. **i** KEGG analysis of DEGs in the control group compared to the CQ group. Only the *SFRP4*-high cluster was classified and analyzed. j Bar plots of the relative percentage of cells in the control and CQ groups. **k** KEGG, GOBP, and GOCC analysis with DEGs of the c4 cluster compared to other clusters. The DEGs used for each analysis had a p value of less than 0.01, and the log two-fold change was >0.1.

To examine the impact of CQ on SEM-type GC organoids at the single-cell level, we conducted a single-nucleus RNA sequencing experiment. We analyzed a sample of SEM-type GC organoids treated with 20 µg/mL CQ (which was not fatal to patients) for 4 days (Fig. 6a). After quality control, a total of 7036 cells remained, which were divided into nine subclusters based on their transcriptomic features (Fig. 6b). Of the nine clusters identified, c0, c1, and c4 exhibited significant expression of *SFRP4*, a biomarker of SEM-type GC (Fig. 6c, d). Thus, we designated these clusters as SFRP4-high SEM-type clusters. These clusters also exhibited high expression of *WNT2B*, a member of the WNT family (Fig. 6c). The WNT signaling pathway is well established as a regulator of cancer stem cell self-renewal and is linked to cancer malignancy through its role in cell growth, differentiation, and metastasis^46,47^. The CellChat analysis revealed that the SFRP4-high SEM-type clusters were the primary source of WNT signals (Fig. 6e). Additionally, the analysis of the intracellular signaling pathway through PROGENy showed a significant increase in the WNT signaling pathway in the SFRP4-high SEM-type clusters (Fig. 6f). Thus, we confirmed that the SFRP4-high SEM-type clusters were stemness-high clusters. ssGSEA revealed that the caveola pathway was highly enriched in the SFRP4-high SEM-type clusters (Fig. 6c) and coenriched with the endocytosis pathway (Fig. 6g). By comparing the differentially expressed genes between the SFRP4-high and SFRP4-low clusters, we found that genes involved in the endocytosis pathway and those related to endosome and lysosome components were upregulated in the SFRP4-high SEM-type clusters. In contrast, genes associated with oxidative phosphorylation and mitochondrial membranes were upregulated in the SFRP4-low clusters, highlighting the clear metabolic differences between the two groups (Fig. 6h and Supplementary Fig. 6a). The responses of the SFRP4-high and SFRP4-low clusters to CQ treatment were investigated. CQ treatment effectively reduced the endocytosis mechanism of the SFRP4-high SEM-type cluster (Fig. 6i and Supplementary Fig. 6b, c). Additionally, CQ treatment significantly reduced the proportion of cluster c4, which represents the SFRP4-high SEM-type clusters in the organoids derived from SEM-type GC patients (Fig. 6j). As expected, pathway analysis of the upregulated genes in each cluster showed that cluster c4 had the highest expression of genes associated with the caveola and endocytosis pathways (Fig. 6k). In conclusion, we distinguished stemness-high clusters within the SEM-type organoids and showed that these clusters were characterized by upregulated endocytosis and caveola pathways. We also confirmed that CQ treatment effectively reduced not only endocytosis but also the proportion of stemness-high clusters in SEM-type GC organoids.

## Discussion

As part of our efforts to understand the differences between tumors, we previously classified GC into five subtypes by analyzing the transcriptomes of the tumors. Among these subtypes, the stem-like subtype was identified as the most malignant, exhibiting the highest drug resistance and the worst prognosis. Notably, the stem-like subtype shares high genetic and molecular similarities with the most malignant subtypes identified in other GC cohorts, such as the EMT-type from ACRG and the GS-type from TCGA-STAD^1^. Due to the various names referring to the subtype with similar mesenchymal traits, which can possibly confuse people, in this study, we defined these subtypes as the SEM (stem-like, GS, EMT, and mesenchymal) type. Although various studies have reported that mesenchymal traits contribute to resistance to therapy^48,49^, medical treatment for GC is still relatively nonspecific. Therefore, in this study, we focused on finding the characteristics and treatment targets specific for SEM-type GC.

In this study, we present CAV1 as a potential marker of SEM-type GC. CAV1 has previously been associated with invasion, metastasis, and resistance to chemotherapy in pancreatic, lung, and bladder cancers^13–18,50^. CAV1 has recently been proposed as a potential biomarker for predicting sensitivity to albumin-binding chemotherapy in breast, pancreatic, and lung cancers^51,52^. While CAV1 is reported to have a role in endocytosis, the importance of CAV1-mediated endocytosis in cancer survival has not been elucidated. In our study, we first demonstrated a vital mechanism for the survival of SEM-type GC cells: CAV1 mediated endocytosis to uptake extracellular proteins, such as albumin. Ultimately, CAV1-positive GC cells showed a significantly higher degree of endocytosis than did CAV1-negative GC cells.

Considering that metastasis is a complex process of cancer cells invading the new environment to thrive in distant organs, cellular uptake of macromolecules provides metabolic advantages for survival in the microenvironment different from the primary site^10,53^. Therefore, endocytosis, which enables scavenging of macromolecules and opportunistic nutrient acquisition, has emerged as a vital feature of malignant cancer cells^8–10^. Likewise, we found that the survival of SEM-type GC cells, which are prone to metastasis, relied heavily on serum while being rarely affected by the depletion of glucose and glutamine, known as essential nutrients for cancer cells^53^. In addition, inhibition of CAV1 was sufficient to cause the death of GC cells. Therefore, we surmise that CAV1-mediated endocytosis increases the chance of survival during the colonization process at the metastatic site, which is a harsh process for cancer cells.

Since both autophagy and endocytosis are lysosome-associated processes^54,55^, we investigated whether CAV1 is also involved in autophagy. We found that SEM-type GC cells had a high enrichment in autophagy-related pathways compared to non-SEM-type GC cells (Supplementary Fig. 7a). However, downregulating CAV1 by siRNA did not affect the protein expression levels of autophagy markers, indicating that CAV1 is unlikely to directly interact with autophagy in SEM-type GC cells (Supplementary Fig. 7b, c). We also compared the effects of the autophagy inhibitors bafilomycin A1 and 3-methyladenine. Bafilomycin a1 disrupts autophagic flux by independently inhibiting V-ATPase-dependent acidification and Ca-P60A/SERCA-dependent autophagosome-lysosome fusion. 3-methyladenine directly inhibits autophagy by blocking autophagosome formation via the inhibition of the class III PI3K complex (Supplementary Fig. 7d). By confirming the IC50 values for each drug, we observed that 3-methyladenine exhibited much lower efficacy in SEM-type GC cells than in non-SEM-type GC cells, despite treating them at significantly higher concentrations than chloroquine or bafilomycin. On the other hand, SEM-type GC cells exhibited much higher sensitivity to bafilomycin, which directly targets lysosomes (Supplementary Fig. 7e). This demonstrates that the inhibition of lysosomes is lethal to SEM-type GC cells, independent of the autophagy mechanism. Therefore, our study focused on CAV1-mediated endocytosis, which takes up extracellular proteins and recycles intracellular components. However, further investigation is necessary to explore the potential interplay between autophagy and endocytosis in SEM-type GC cells.

Assuming that targeting endocytosis would be fatal for SEM-type GC cells, we tested the effectiveness of chloroquine (CQ), an anti-malarial drug known for its ability to inhibit endocytosis and lysosome function^25,26^. Lysosome-mediated metabolism has been found to be important for cancer cells in rapidly growing or undernourished environments^41^, which has indicated that CQ could be a candidate for anticancer drugs. As a result, CQ has been studied as an autolysosomal inhibitor, a p53 pathway activator, and an apoptosis inducer in pancreatic cancer, lymphoma, and glioma, respectively^27–29^. However, none of the clinical trials regarding CQ as an anticancer drug have been successful^30–32^. We speculate that the failure of clinical trials regarding CQ as an anticancer drug may have come from the lack of consideration of tumor heterogeneity. By introducing a biomarker such as CAV1, we hope to screen GC patients who may potentially benefit from CQ treatment. Our data showed that CQ treatment was significantly more effective for CAV1-positive SEM-type GC cells than for CAV1-negative non-SEM-type GC cells. We confirmed that CQ effectively blocked the engulfment of macromolecules and induced significant damage to lysosomes and mitochondria in GC cells. Interestingly, these effects occurred in both SEM-type and non-SEM-type GC cells but were much more fatal to SEM-type GC cells.

Moreover, to evaluate the effect of CQ under more physiological conditions, we generated patient-derived organoids and mouse xenograft models. We confirmed that CQ treatment significantly reduced the growth of SEM-type GC organoids and in vivo tumor size. Since patients with SEM-type GC receive little benefit from conventional chemotherapy, this tumor suppression effect of CQ is noteworthy. In addition, through analysis of snRNA sequencing data, we identified the SFRP4-high clusters as representing stem-high clusters within the SEM-type organoid. These clusters were characterized by high levels of endocytosis and the caveola pathway, making them highly responsive to CQ. Therefore, we propose the use of CQ in combination with existing anticancer drugs as a promising solution for effectively treating SEM-type gastric cancer and overcoming its limitations.

SEM-type GC tumors show high stemness, resulting in a high degree of cellular heterogeneity within the tumors. Due to this intratumoral heterogeneity, it is important to evaluate the performance of anticancer drugs at single-cell resolutions, aiming to target cells that are the primary drivers of tumor aggressiveness. Here, we conducted single-nucleus RNA sequencing with and without CQ treatment. CQ treatment successfully targeted stemness-high cell clusters with high self-renewal capabilities, higher metastatic potential, and resistance to chemotherapy. These findings suggest that CQ is a promising therapeutic regimen for targeting chemoresistant SEM-type GC cells.

In conclusion, we demonstrated that CAV1 serves as a biomarker for SEM-type GC and that CAV1-mediated endocytosis plays a critical role in the uptake of extracellular proteins as a nutrient. Extracellular protein is an essential energy source for SEM-type GC cells, making CQ a potential anticancer drug for chemoresistant GC patients. We found that CQ greatly reduced the tumor burden in vivo and had an antiproliferative effect on patient-derived organoids from SEM-type GC tumors. Our study suggests that CQ, whose safety has already been proven, will be a therapeutic alternative for chemoresistant SEM-type GC patients.

### Supplementary information

Supplementary data

Supplementary Video 1

## Data Availability

All data that support the findings of this study are available from the authors upon reasonable request.
