# Supplementary material for: Caveolin-1 mediates the utilization of extracellular proteins for survival in refractory gastric cancer
Source: Exp Mol Med. 2023 Nov 2;55(11):2461–72. doi: 10.1038/s12276-023-01109-7 (PMC10689497; doi:10.1038/s12276-023-01109-7)
Supplement: Supplementary file 1 — Supplementary data [file 12276_2023_1109_MOESM1_ESM.pdf]

1. Supplementary Methods

TCGA gastric cancer dataset.

TCGA provided pre-processed RNA-seq data for all available datasets from TCGA STAD in August 2019, which was downloaded using R package TCGAbiolinks (GDCquery and GDCdownload functions). Preprocessing was done with TCGAanalyze_Preprocessing function using a 0.6 Spearman correlation cut-off. Normalization was done with TCGAAnalyze_Normalization function for GC-content and library size. Pearson's correlation coefficient was calculated with ggplot2.

Bioinformatics.

The SEEK database was used to identify co-expressed genes with CAV1 in human GC datasets (37 datasets). Gene set enrichment analysis (GSEA) was performed using patient microarray data in the Yonsei cohort and RNA-seq data in cell lines and tumors. Public microarray data analysis was obtained from Gene Expression Omnibus in the NCBI database. The pathway enrichment score was computed with the GSVA function in R, and hierarchical clustering was used to draw a heatmap for the expression of CAV1 and its co-expressed genes in TCGA STAD patients and Caveolae complex genes in GC cell lines. A volcano plot was created based on DEG sorted by fold change and p-value in microarray data from human patients in the Yonsei cohort, visualized by log2(FC) and adjusted p-value

GC cell lines RNA Sequencing.

Total RNA was extracted from 29 gastric cancer cell lines using a RNeasy Plus Mini Kit (QIAGEN, DE) and mRNA-focused libraries were generated with TruSeq RNA Sample Prep kit v2 (Illumina, USA). HiSeq 2500 platform was used to sequence the libraries, generating at least 40 million paired-end reads of 100bp per sample. The reads were aligned to the reference genome and FPKM values were calculated using the TopHat-Cufflinks pipeline. RNA-seq data were deposited in NCBI SRA (#SRP078289).

siRNA intervention.

To knockdown CAV1 in HS746T, caveolin-1 siRNA (h) (sc-29241, Santa Cruz, USA) and control siRNA (Santa Cruz, USA) were transfected with Viromer BLUE transfection reagents (OriGene, USA) or electroporation for other GC cell lines. The efficiency of silencing was evaluated by western blotting after 48hr.

Plasmid transfection.

To overexpress CAV1 in GC cells, CAV1-mCherry vector (27705, Addgene, USA) was introduced, with pRSET-BmCherry (Addgene, USA) serving as the control. Viromer RED transfection reagents (OriGene, USA) were used for HS746T cells according to the manufacturer's instructions, and microspore electroporation was used for other

cell lines.

RNA extraction and Real-time quantitative PCR analysis.

To analyze mRNA expression levels (n=3/group), total RNA was isolated from GC cells using TRIzol reagent (Invitrogen, USA) and cDNA was synthesized using SuperScript reverse transcriptase II (Invitrogen, USA). RT-qPCR was performed with SYBR Green PCR Master Mix (Applied Biosystems) using ABI PRISM 7300 RT-PCR system (Applied Biosystems) and data were normalized to 18S ribosomal RNA expression level using the delta delta Ct method. Primers are as follows: 18S_S, 5'- GTA ACC CGT TGA ACC CCA TTT -3'; 18S_AS, 5'- CCA TCC AAT CGG TAG TAG CG -3', CAV1_S, 5'- CCA AGG AGA TCG ACC TGG TCA A -3'; CAV1_AS, 5'- GCC GTC AAA ACT GTG TGT CCC T-3'.

Western blotting.

Proteins were extracted from cells using 1% SDS-containing whole cell lysis buffer, and quantified using BCA assay kit (Pierce, USA). Equal amounts of protein were loaded, separated by SDS-PAGE, and transferred onto nitrocellulose membranes. Membranes were blocked with 5% skim milk for 1h at RT and incubated overnight at 4°C with primary antibodies against β-actin (sc-47778, Santa Cruz, USA), Caveolin-1(ab2910, Abcam, UK), GAPDH (2118S, CST, USA), and LAMP-1 (sc-20011, Santacruz, USA), diluted in 5% BSA in PBS. After washing, membranes were incubated with horseradish peroxidase-labeled secondary antibodies for 1h at RT.

CAV1-knockdown cells RNA sequencing.

Two days after transfection of HS746T with control or CAV1-siRNA (n=2/group), total RNA was isolated using TRIzol reagent (Invitrogen, USA), and the RNA concentration was determined using Quant-IT RiboGreen (Invitrogen, USA). TapeStation RNA screentape (Agilent, USA) was used to assess RNA purity. High-quality RNA samples with RIN greater than 7.0 were used for RNA library construction using the Illumina TruSeq RNA sample Preparation Kit (Illumina, San Diego, CA, USA), with mRNA purification performed using poly-T-attached magnetic beads. Libraries were quantified using the KAPA library quantification kit and qualified using TapeStation D1000 ScreenTape (Agilent, Santa Clara, CA, USA). Indexed libraries were sequenced using Illumina NovaSeq with paired-end (2×100 bp) sequencing by Macrogen Incorporated.

Immunofluorescence.

Approximately 1.5×10^5 cells were seeded into 12-well plates with glass coverslips (n=2/group). After each experiment, the cells were fixed, permeabilized, and blocked before incubating with anti-Caveolin-1 antibody (Abcam, UK) and LAMP-1 antibody (Santa Cruz, USA). Fluorescent secondary antibody was then added, and the samples were mounted with DAPI and imaged using a Zeiss LSM-780 microscope. Airyscan processing was

performed using ZEN2.3 software (Carl Zeiss).

Live cell imaging.

1.5×10^5 GC cells were seeded into Ibidi Glass Bottom Dish 35 mm (n=2/group). After experiment, the cells were treated with Lysotracker Blue, Mito tracker, ER-tracker, and Acridine orange (Invitrogen, USA) respectively for 30min at 37°C. Imaging and analysis were conducted with Zeiss LSM-780 microscope and Airyscan processing using ZEN2.3 software (Carl Zeiss).

Measuring endocytosis.

70kDa TMR-DEX (Invitrogen, USA) was used to measure endocytic uptake. After the experiment, cells were serum-starved for 12h and then exposed to TMR-DEX (1mg/ml) for 30min at 37°C. Confocal or live-cell imaging was used for analysis.

Oxygen consumption rate measurement.

GC cells were seeded onto Seahorse XFp assay plates at 70-80% confluency (n=3/group). Seahorse XF mito stress kit (Agilent, USA) was used to evaluate the OCR of cells pre-treated with 100μM BSA (Sigma, USA) in serum-free condition for 1h.

Single-nucleus RNA sequencing analysis.

Nuclei were isolated via Flow Cytometry from a homogenized frozen sample and used to create a barcoded cDNA library with 10X Genomics Chromium Instrument and cDNA synthesis kit (10x Genomics: Chromium Next GEM Automated Single Cell 3′ Library and Gel Bead Kit v3.1). The quality of the cDNA library was assessed using an Agilent Bioanalyzer. We ran two paired-end 100bp Flow Cells on an Illumina Novaseq 6000 with the cDNA library, aligning the demultiplexed fastq files to the mouse genome using 10X Genomics Cellranger with the option --force-cells 2000. The read10X function of Seurat[1] was used to load the Cellranger output, and the package pipeline was used for gene expression and cluster detection. For quality control, we removed cells with 10% high or low feature counts and those with 20% higher mitochondrial gene percentages. We normalized and scaled using the default settings. To remove batch effects among samples Seurat's FindIntegrationAnchors and IntegrateData commands were used. To construct the shared nearest-neighbor graph on UMAP coordinates, we utilized the FindNeighbors function and performed SNN modularity optimization on the graph using the FindClusters function. Dimensional reduction was performed with the RunUMAP function. We applied the expression recovery algorithm, ALRA, to further analyze cell populations with restored expression using the RunALRA command of SeuratWrappers package with default settings. To compare gene expression between groups, Seurat's FindMarkers or FindAllMarkers function was used, employing the Wilcoxon Rank Sum test with default settings.

Genes with logFC greater than 0.10 and a p-value less than 0.01 were retained. Pathway analysis of the resulting genes was conducted using an R package PROGENy (1.17.3)[2], escape (1.8.0)[3], and clusterProfiler (4.0)[4]. Only pathways with adjusted p-values lower than 0.05 were selected. Cell-cell communication was analyzed using the R package CellChat (1.6.0)[5].

HS746T-GFP cell line.

Puromycin-resistant HS746T-GFP cells were generated by incubating the cells in media containing lentivirus produced by co-transfecting pLKO.1-puro-CMV-TurboGFP, pMD2.G, and psPAX2 in 293T human embryonic kidney cells.

IVIS imaging.

iPM mouse models were imaged with IVIS spectrum imaging systems (Caliper Life Sciences) under respiratory anesthesia with 2% isoflurane. Living Imaging Software version 4.4 (Caliper Life Science) was used to analyze luminescence images, measure photon emission within a region of interest, and calculate radiant efficiency ([photons/sec/cm2/sr]/[μW/cm2]).

References

1 Hao, Y. *et al.* Integrated analysis of multimodal single-cell data. *Cell* **184**, 3573-3587 e3529, doi:10.1016/j.cell.2021.04.048 (2021).

2 Schubert, M. *et al.* Perturbation-response genes reveal signaling footprints in cancer gene expression. *Nat Commun* **9**, 20, doi:10.1038/s41467-017-02391-6 (2018).

3 Borcherding, N. *et al.* Mapping the immune environment in clear cell renal carcinoma by single-cell genomics. *Commun Biol* **4**, 122, doi:10.1038/s42003-020-01625-6 (2021).

4 Wu, T. *et al.* clusterProfiler 4.0: A universal enrichment tool for interpreting omics data. *Innovation (Camb)* **2**, 100141, doi:10.1016/j.xinn.2021.100141 (2021).

5 Jin, S. *et al.* Inference and analysis of cell-cell communication using CellChat. *Nat Commun* **12**, 1088, doi:10.1038/s41467-021-21246-9 (2021).

2. Supplementary figures

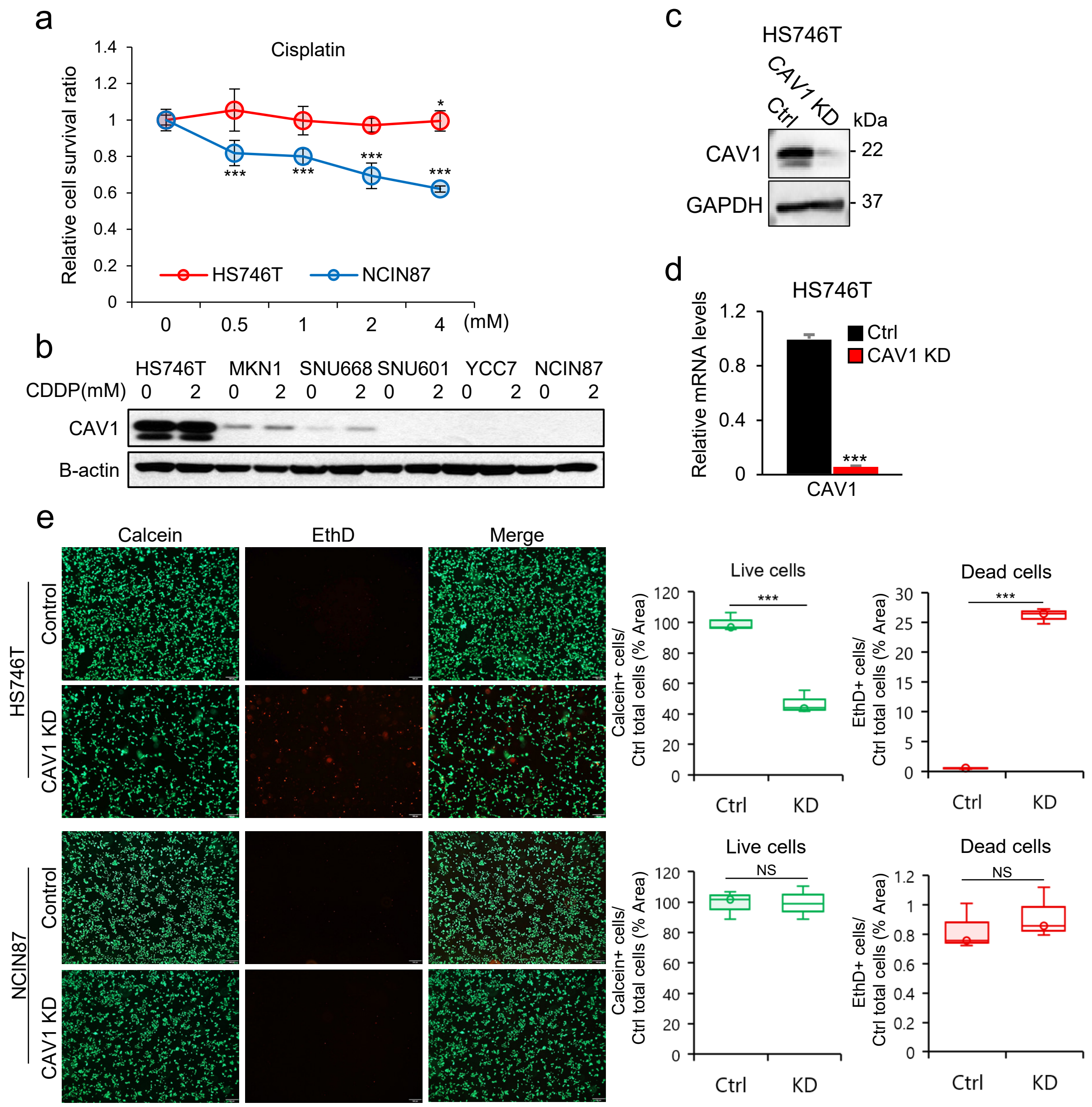

Supplementary Fig. 1 Inhibition of CAV1 is fatal to SEM-type GC cells. (a) The IC50 values of cisplatin (CDDP) treatment for HS746T(red) and NCIN87(blue), respectively. (b) Expression of CAV1 in HS746T after CAV1-knockdown with siRNA at the protein levels (c and d) Expression of CAV1 in HS746T after knockdown with siRNA at the protein levels (c) and the relative mRNA levels (d). (e) Fluorescence imaging of calcein AM and EthD-1 (LIVE/DEAD) stained HS746T and NCIN87 cells after CAV1-knockdown for 48hr (left). The area positive for calcein staining over a total area demonstrated a significant decrease in cell viability only in CAV1 knocked-down SEM-type GC cells (right). Data represent mean ± SD. ** $p < 0.01$; *** $p < 0.001$; two-tailed t test.

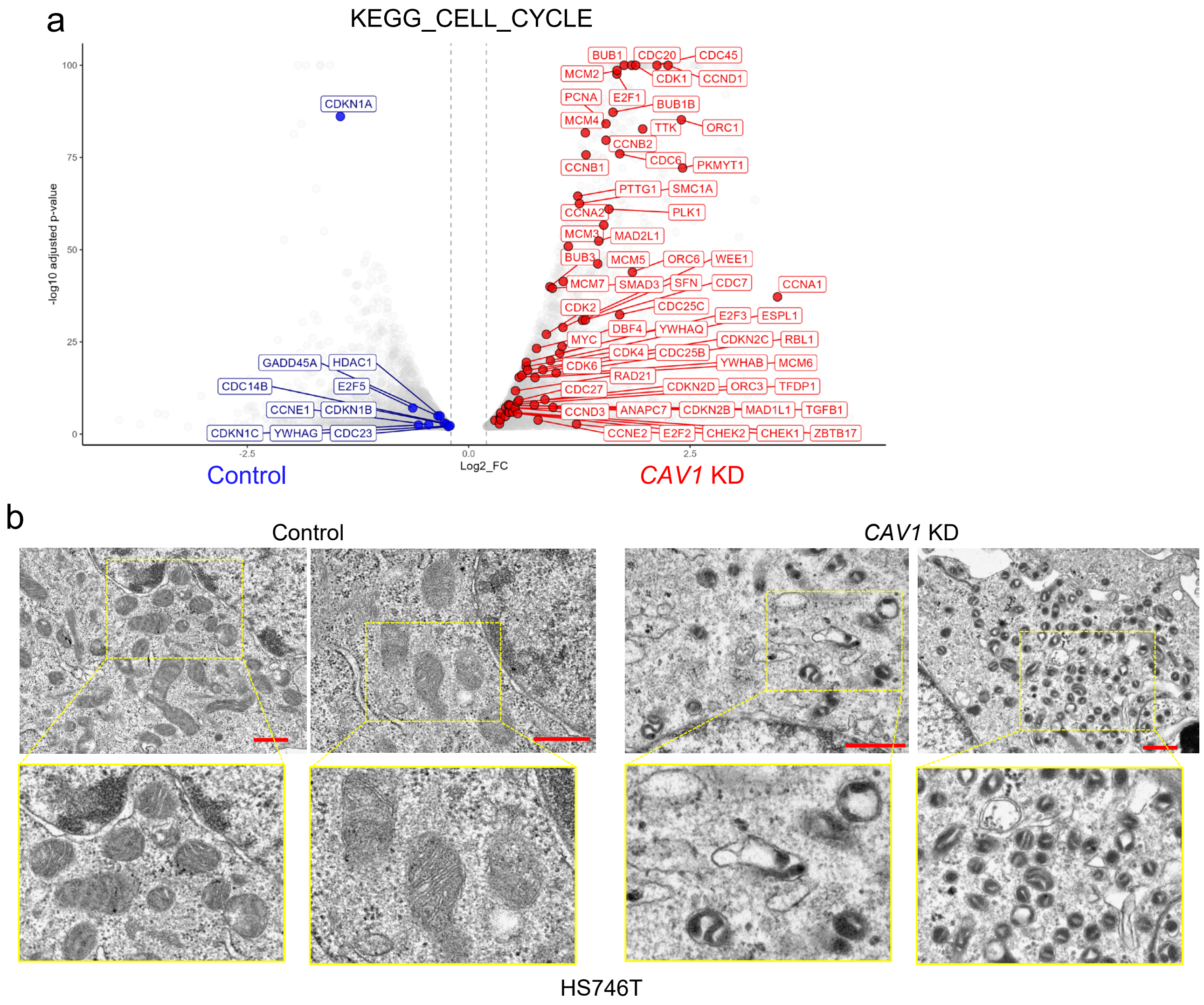

Supplementary Fig. 2 Inhibition of CAV1 is fatal to SEM-type GC cells. (a)Volcano plot representing gene sets of KEGG_CELL_CYCLE in the DEGs of HS746T after CAV1 knockdown (blue; down-regulated, red; up-regulated, log2 fold change>0.2, adjusted p-value<0.01) (b) TEM images showing damaged mitochondria in CAV1-knockdowned HS746T. N; nucleus, arrow; mitochondria. Scale bar, 1µm.

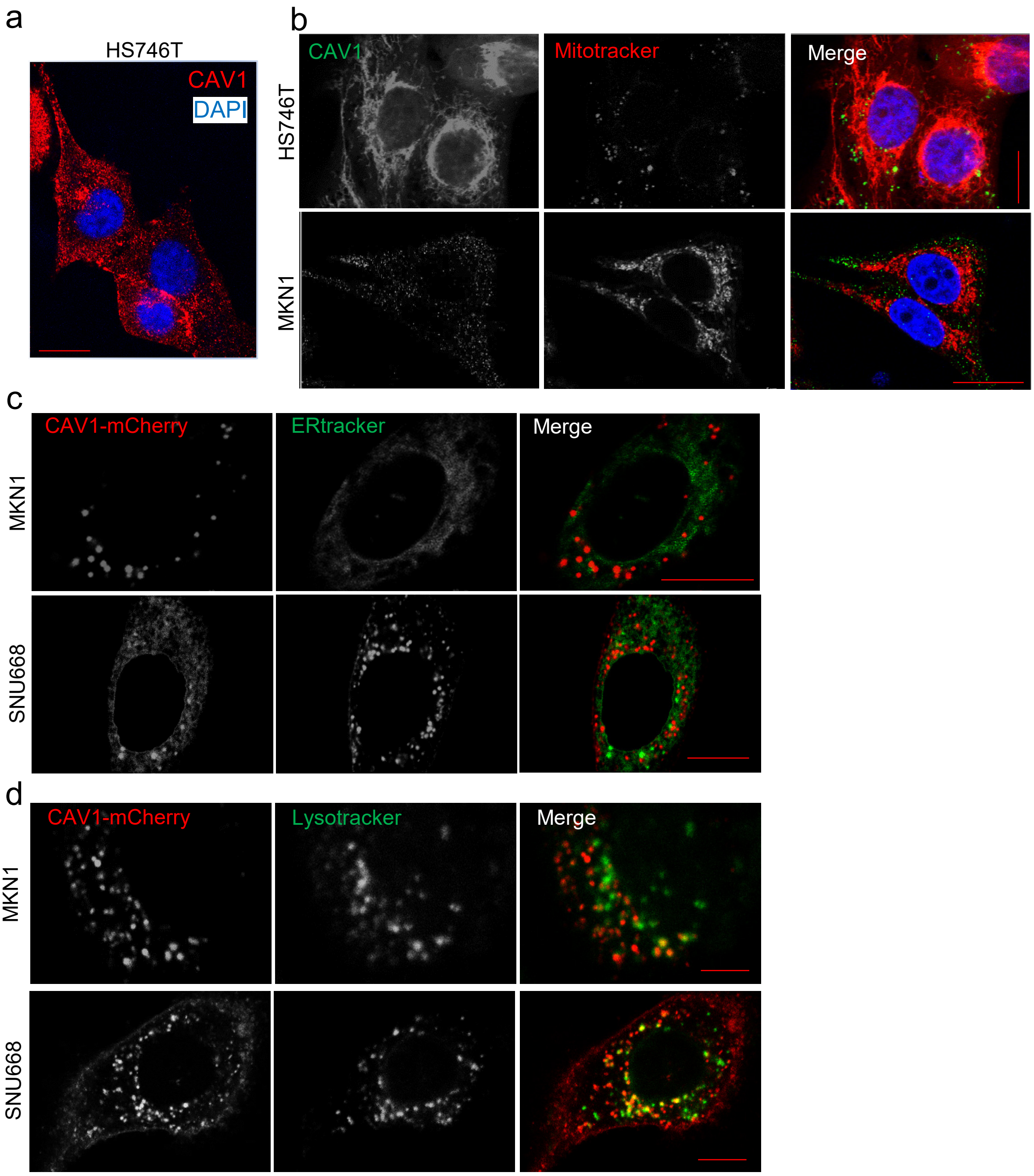

Supplementary Fig. 3 CAV1 is located in lysosome in SEM-type GC cells. (a) A Confocal image of CAV1(red) and DAPI (blue) in HS746T. Scale bars, 20μm. (b) Confocal images of HS746T and MKN1 expressing mCherry-CAV1(left), Mito-tracker(middle). Merged images are a two-channel combination of original image pairs (mCherry-cav1; green, Mito-tracker; red, DAPI; blue, right). Scale bar, 20μm. (c) Live cell images of SNU668 and MKN1 expressing mCherry-CAV1(left), ER-tracker(middle). Merged images (mCherry-CAV1; red, ER-tracker; green, right). Scale bar 5μm,10μm respectively. (d) Live cell images of SNU668 and MKN1 expressing mCherry-CAV1(left), lysotracker (middle) and. Merged images (mCherry-CAV1; green, lysotracker; red, right). Scale bar 20μm and 5μm, respectively.

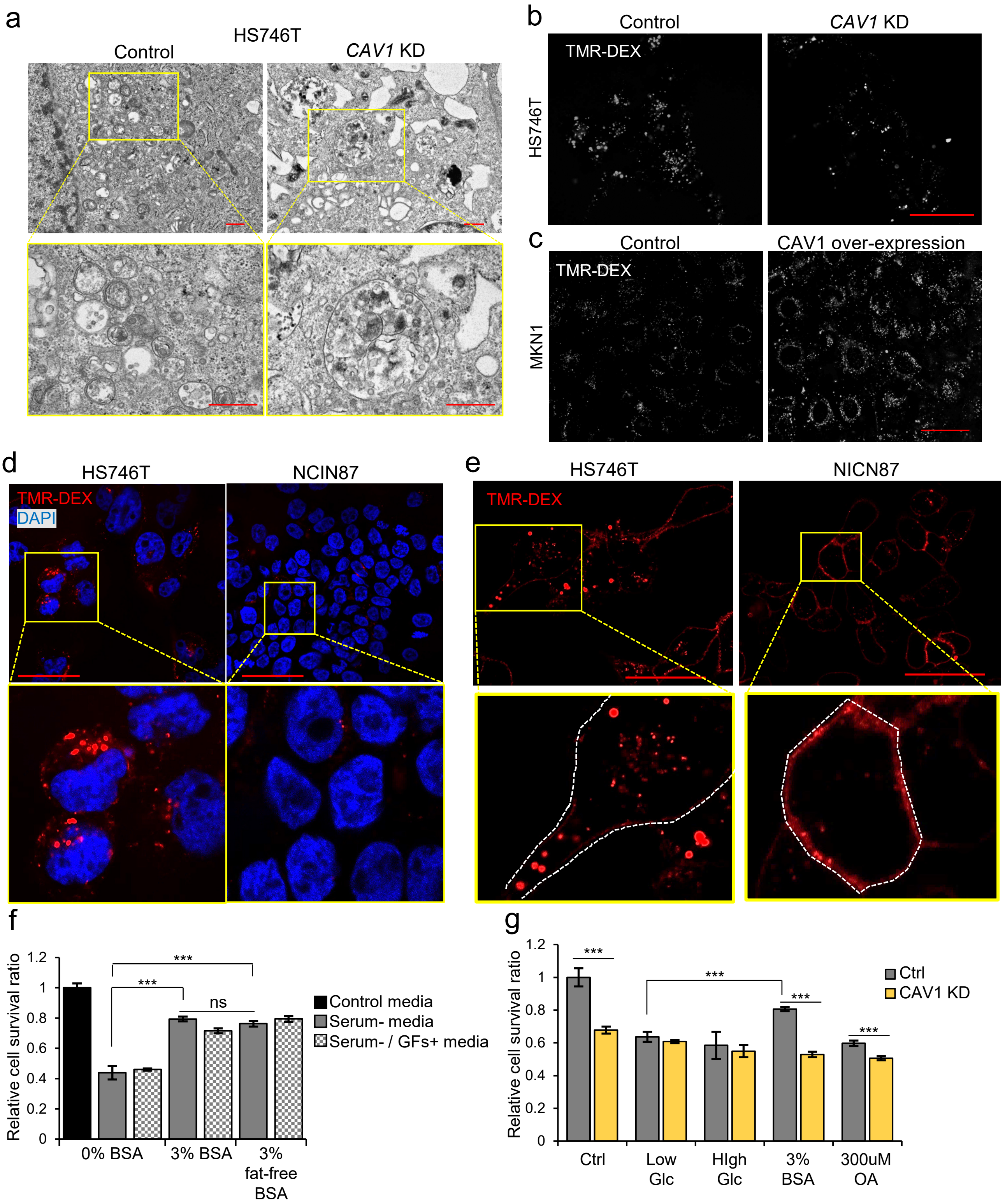

Supplementary Fig. 4 CAV1-mediated endocytosis is up-regulated in SEM-type GC cells and is effectively regressed by lysosome inhibitors. (a) TEM images showing damaged lysosome in CAV1-knockdowned HS746T. N; nucleus, arrow; mitochondria. Scale bar, 1μm. (b and c) TMR-DEX uptake assay of (b) CAV1-knockdowned HS746T and (c) CAV1-overexpressed MKN1. Scale bars, 50μm. (n=2 /group). (d and e) Confocal images of HS746T and NICN87 after TMR-DEX uptake assay. The cells were imaged fixed (d) or alive (e) (TMR-DEX; red, DAPI; blue). Scale bars, 50μm. (f) Bar plot for the survival rates of cells cultured in serum-free media with or without the addition of growth factors (GFs), and with the presence

or absence of 3% BSA or lipid-free BSA, respectively. (g) Bar plot of the relative percentage of live cells in the control and CAV1-KD groups in each nutrient condition; control DMEM, Low Glc (1g/L glucose, 4mM glutamine, 1% FBS), high Glc (4.5 g/L glucose, 4mM glutamine, 1% FBS), Low Glc + 3% BSA, and Low Glc + 300μM BSA-conjugated oleic acid, respectively. Data represent mean ± SD. ** $p < 0.01$; *** $p < 0.01$; two-tailed t test.

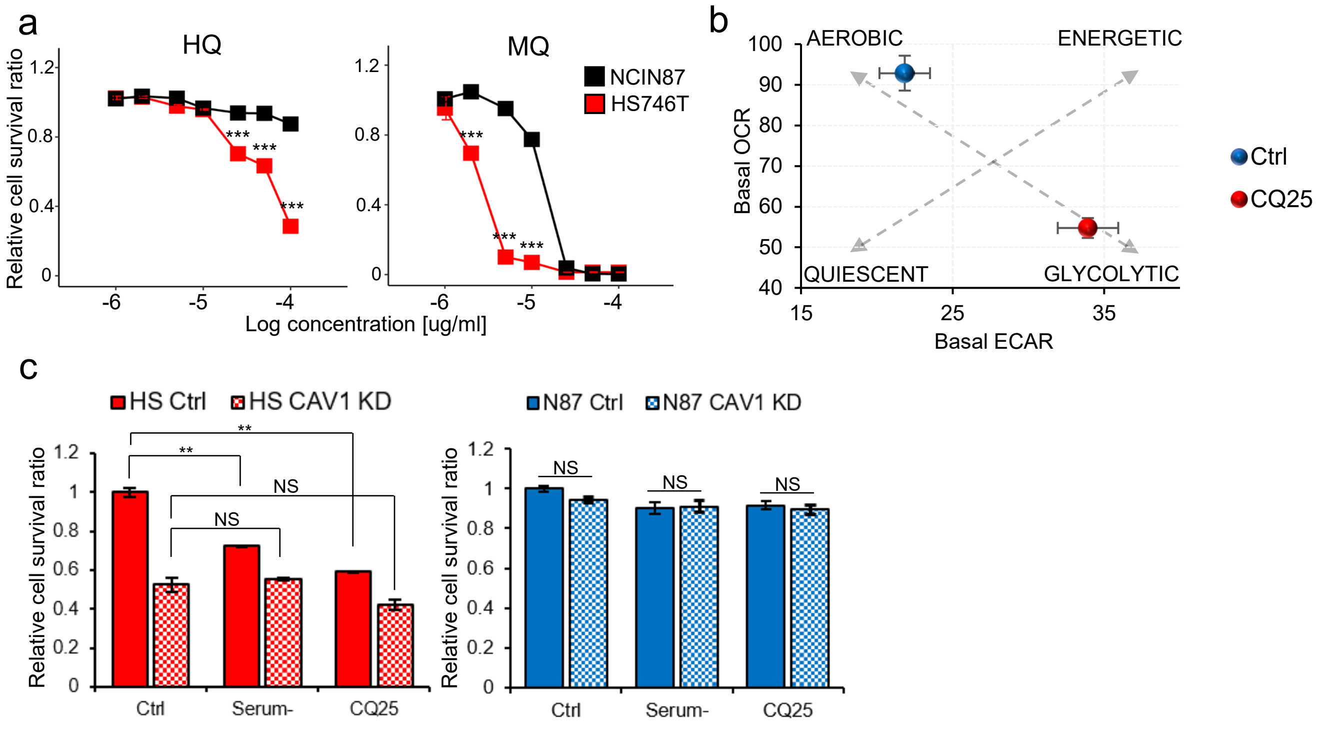

Supplementary Fig. 5 CAV1-mediated endocytosis is up-regulated in SEM-type GC cells and is effectively regressed by lysosome inhibitors. (a) The IC50 values of Hydroxy-chloroquine (HQ), and Mefloquine (MQ) treatment for HS746T(red) and NCIN87(black), respectively. (b) The energy map for HS746T treated with CQ 25µg/ml reveals that cells exhibit a metabolic shift from an aerobic to a glycolytic phenotype. The dotted lines represent the aerobic-glycolytic axis and the quiescent-energetic axis. Data are expressed as mean ± SEM (n = 4 for each mean depicted). Raw data were analyzed using Seahorse Wave Software (version 2.3.0.19). (c) Bar plots depict the survival rates of CAV1-KD cells cultured in serum-free media or treated with CQ 25µg/ml for 48hr, respectively. Data represent mean ± SD. ** $p < 0.01$; *** $p < 0.001$; two-tailed t test.

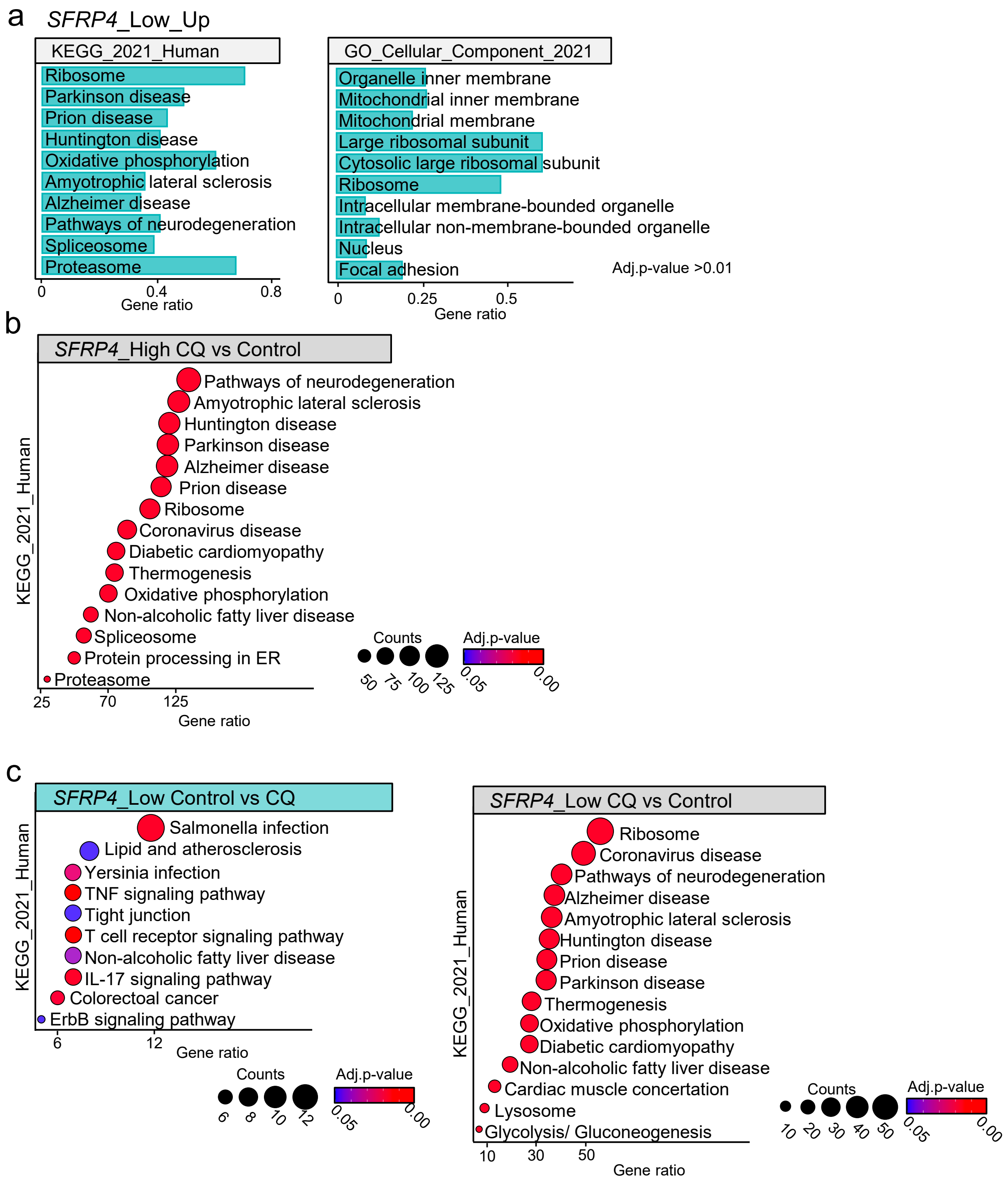

Supplementary Fig. 6 Effect of CQ on SFRP4-high and –low clusters in SEM-type GC organoid, respectively. (a) KEGG and GOCC analysis with DEGs of SFRP4-low compared to –high cluster (b) KEGG analysis with DEGs of control group compared to CQ. SFRP4-high cluster were classified and analyzed. (c) KEGG analysis with DEGs of CQ group compared to Control. SFRP4-low cluster were classified and analyzed. The DEGs used for each analysis had a p-value of less than 0.01, and the log2 fold change was greater than 0.1.

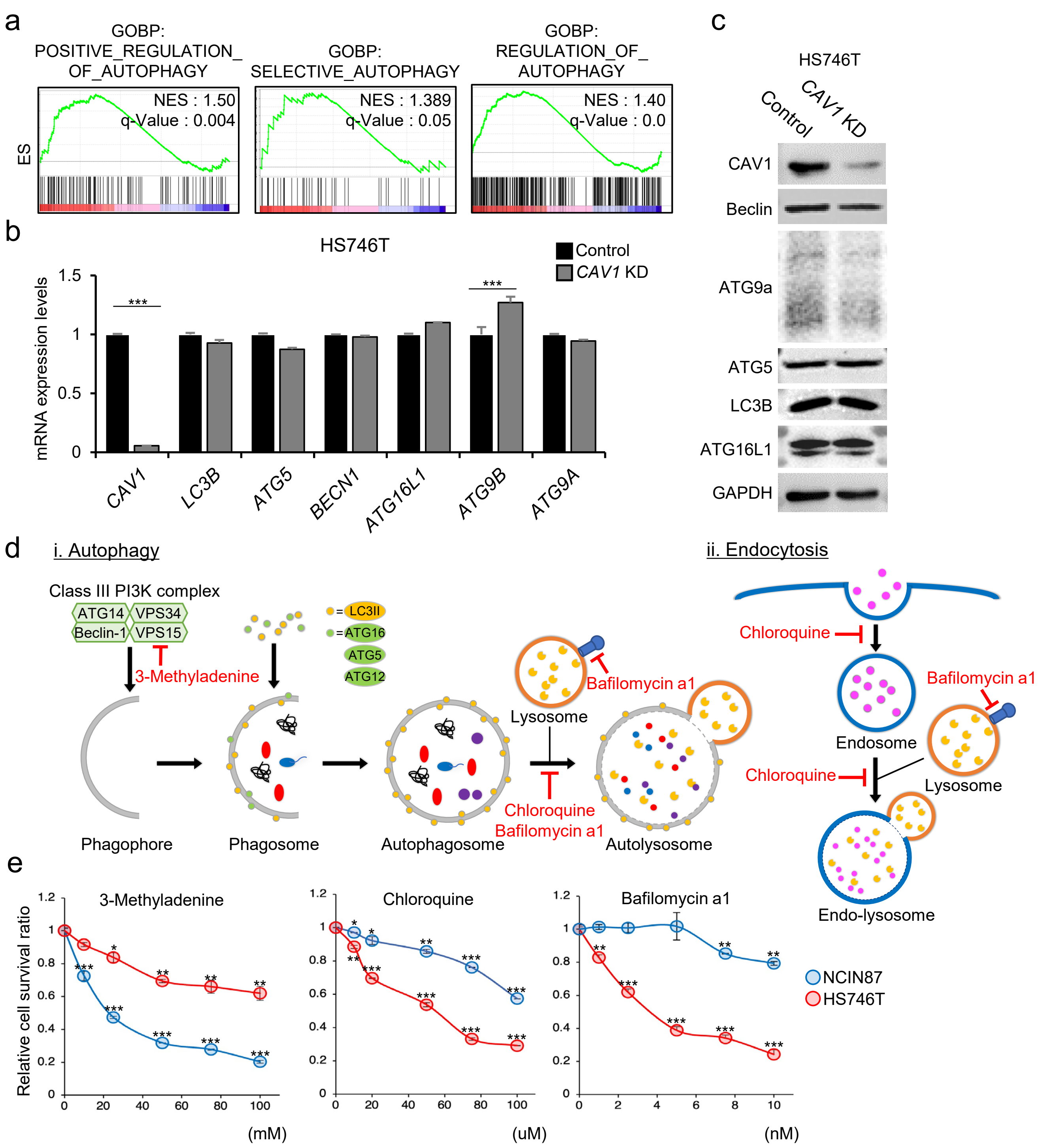

Supplementary Fig. 7 The role of CAV1 is not related to autophagy, but to endocytosis. (a) Gene Set Enrichment Analysis (GSEA) for GOBP_POSITIVE_REGULATION_OF_AUTOPHAGY, GOBP_SELECTIVE_AUTOPHAGY, and REGULATION_OF_AUTOPHAGY from MsigDB v7.0 in SEM type GC cells compared to non-SEM type GC cells (b and c) Expression of CAV1 in HS746T after knockdown with siRNA at the relative mRNA levels (b) and the protein levels (c). (d) Schema depicting mechanism of 3-Methyladenine, Chloroquine, and Bafilomycin a1 on autophagy and endocytosis (e) The IC50 values of each drug for HS746T(red) and NCIN87(blue), respectively. Data represent mean ± SD. ** $p < 0.01$; *** $p < 0.001$; two-tailed t test.
